# Application of an algorithm to analyze patterns of intermittent oral corticosteroid use in asthma

**DOI:** 10.1038/s41533-023-00331-0

**Published:** 2023-03-04

**Authors:** John Haughney, Trung N. Tran, Heath Heatley, Arnaud Bourdin, Andrew Menzies-Gow, David J. Jackson, Ekaterina Maslova, Jatin Chapaneri, Derek Skinner, Victoria Carter, Jeffrey Shi Kai Chan, David Price

**Affiliations:** 1 NHS Clinical Research Facilities, Glasgow, UK; 2 AstraZeneca, Gaithersburg, MD USA; 3 Observational and Pragmatic Research Institute, Midview City, Singapore; 4 Department of Respiratory Diseases, Montpellier University Hospitals, Arnaud de Villeneuve Hospital, Montpellier, France; 5 UK Severe Asthma Network and National Registry, Royal Brompton & Harefield Hospitals, London, UK; 6 UK Severe Asthma Network and National Registry, Guy’s and St Thomas’ NHS Trust and Division of Asthma, Allergy & Lung Biology, King’s College London, London, UK; 7 AstraZeneca, Cambridge, UK; 8 Centre of Academic Primary Care, Division of Applied Health Sciences, University of Aberdeen, Aberdeen, UK

**Keywords:** Asthma, Preventive medicine

## Abstract

An algorithm to describe patterns of intermittent oral corticosteroid use in the UK (*n* = 476,167) found that one-third of patients receiving intermittent oral corticosteroids for asthma only had short gaps (<90 days) between oral corticosteroid prescriptions sometime during follow-up. The increasing frequency pattern was more likely in patients with greater asthma severity and with more short-acting β_2_-agonist use at baseline. Our approach may provide a clinically relevant representation of intermittent oral corticosteroid use in asthma.

## Introduction

Despite increasingly widespread use of newer treatment regimens, oral corticosteroids (OCS) continue to be used for acute exacerbations of asthma and as a daily therapy for severe refractory asthma^1^. Increasing cumulative exposure to OCS is associated with an increased risk of developing acute and chronic OCS-related adverse outcomes^2^.

There is currently no universal definition for either intermittent or long-term OCS use, making it challenging to differentiate these patterns of use. Previous attempts to categorize OCS use have been based on aggregating prescriptions over a specific time period and have investigated a mixture of intermittent and long-term OCS use^3–5^.

There are potentially many different patterns of intermittent OCS use, which may have varying impacts on the risk of developing adverse events (AEs)^2,6,7^. Characterizing these patterns of intermittent use is a first step towards understanding their association with risk of AEs over time.

This study aimed to describe and quantify patterns of intermittent OCS prescriptions in patients with asthma according to a novel algorithm identifying intermittent prescriptions. We also aimed to assess the distribution of these patterns by Global Initiative for Asthma (GINA) treatment step, short-acting β_2_-agonist (SABA) use and age.

## Methods

### Study design and patients

This was an historical UK cohort study using data from the Optimum Patient Care Research Database (OPCRD)^8^ and Clinical Practice Research Datalink (CPRD)^9^ of electronic medical records between 2008 and 2019. The study was registered at the European Network of Centers for Pharmacoepidemiology and Pharmacovigilance (ENCePP; registration number: EUPAS37065). The algorithm used to identify intermittent OCS prescriptions, described in detail below, was developed based on OCS prescriptions extracted from OPCRD. Description of longitudinal patterns of intermittent OCS use overall and by GINA treatment step and SABA use was based on data from both OPCRD and CPRD as an indirect validation against disease severity and control, respectively.

Patients aged ≥4 years with ≥1 asthma event (medication, asthma consultations and/or asthma diagnosis) within 3 months of the first OCS prescription (Index date) were included in the analysis. Patients were included in the OCS arm if they had a prescription of an OCS with a concurrent (within 3 months) asthma event defined as an asthma quality and outcomes framework (QOF) diagnosis or asthma QOF prescription. Patients were included in the non-OCS arm if they had no OCS prescription at any time. Patients in both arms had ≥12 months’ baseline period (prior to index date). Patients aged ≥18 years were excluded if they were ever diagnosed with the following chronic conditions, treated with OCS: ankylosing spondylitis, Sjogren’s syndrome, systemic lupus erythematosus, temporal arteritis, ulcerative colitis, psoriatic arthritis, multiple sclerosis, polymyalgia rheumatica, Crohn’s disease or cancer of respiratory system. Patients aged ≥4–<18 years were excluded based on the analysis of concurrent OCS and diagnoses.

**Fig. 1:**
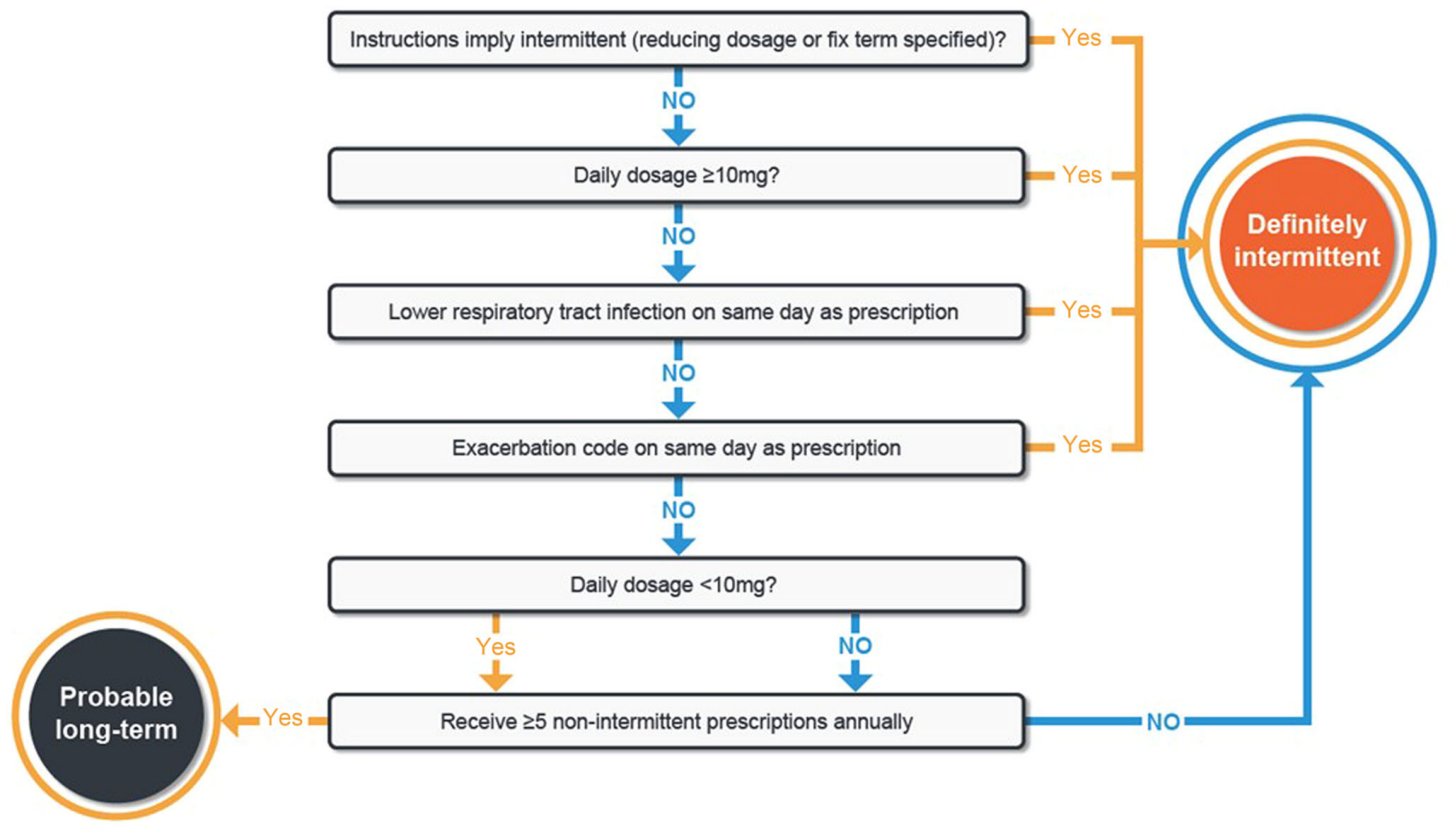
OPRI OCS algorithm to classify intermittent and long-term OCS use*. *OCS doses represent prednisolone equivalent.

Patients’ OCS prescriptions were identified as intermittent (repeated [≥2/year] acute courses) according to the algorithm shown in Fig. 1. Expert respiratory clinicians were involved in the development of this algorithm, which used information associated to the OCS prescriptions and recommendations by the National Institute for Health and Care Excellence (NICE) guideline^10^ to determine whether they were likely an intermittent or a maintenance prescription. This was done in a stepwise approach, starting with the most definitive information. The initial step questioned whether the prescription instructions implied intermittent prescribing. For prescriptions that did not enable a decision using the dosing instructions, the next most definitive information was used, which was the daily dose. Following that, concurrent lower respiratory tract infections or asthma exacerbations, and annual number of non-intermittent prescriptions were used. In this manner, the algorithm aimed to maximize specificity (i.e., excluding any prescription that might potentially imply long-term OCS use).

There were no criteria or threshold specified to define a course of OCS prescription, and exposures were considered as one script regardless of dose. Doses of different types of OCS exposures were converted into prednisolone equivalents using the defined daily dose (DDD) obtained from the Anatomical Therapeutic Chemical (ATC)/DDD classification system.

This study was approved by the Anonymised Data Ethics & Protocol Transparency (ADEPT) Committee^11^ (ADEPT1120) and the Independent Scientific Advisory Committee (ISAC 20_000071). The basis of this approval is that data transferred and held in OPCRD are fully anonymous, patient level information having been de-identified ‘at source’. Patient consent is considered under an opt-out basis within their general practice electronic health record (EHR). Options available in the EHR system allow for selection of an individual patient and for that patient to be flagged as opting out of data sharing and OPCRD extraction. If this option is selected, the patient’s data will not be extracted by OPCRD for research or for data linkage. OPCRD also reviews and respects clinical codes that flag patient objections to their data being used for various purposes by not collecting these data (further details are available here: https://opcrd.co.uk/our-database/data-access-governance/).

### Treatment group categorization

OCS prescription patterns were classified into three distinct use patterns, based on the spacing of OCS bursts across patients’ entire follow-up: **once-only** – patients with only one OCS prescription ever in their electronic medical records; **sporadic** – prescriptions with gaps ≥365 days; **infrequent** – prescriptions with gaps 182–364 days; **moderately frequent** – prescriptions with gaps 90–181 days; and **frequent** – prescriptions with gaps <90 days. For instance, experiencing ≥2 courses within four weeks (<90 days), regardless of temporal gaps between prescription clusters, was defined as ‘frequent’ use (Supplementary Fig. 1). During follow-up, patients could have one or a mixture of the patterns listed above.

### Treatment group stratifications

Intermittent OCS use pattern was stratified by GINA 2020 treatment steps (no asthma medication and Steps 1–5)^12^ and SABA use (0, 1–2 and ≥3 fills) assessed in the 12 months prior to the first OCS prescription recorded in the database, which for some patients could represent their first presentation for asthma. Intermittent OCS use pattern was also stratified by age (children [≥4–<12 years], adolescents [≥12–<18 years] and adults [≥18 years]).

### Statistical analysis

Summary statistics were provided for the decision step of the OPRI OCS algorithm.

Sequence and pattern analyses were used to describe OCS prescribing patterns and determine OCS prescribing categories. Statistical significance (Chi-square test) was defined at *p* < 0.05.

### Reporting summary

Further information on research design is available in the Nature Research Reporting Summary linked to this article.

## Results

Of 2,130,881 patients receiving OCS prescriptions for any condition in both CPRD and OPCRD, 476,167 patients met the inclusion criteria, had only intermittent OCS use, and were either in a single category of OCS use (266,562; 56.0%) or had mixed prescribing patterns (209,605; 44.0%). Of the included 476,167 patients, 44.3% were male and mean age (standard deviation) was 38.1 (22.4) years.

Overall, 198,422 (41.7%) were classified as having once-only OCS, 65,632 (13.8%) as sporadic only, 33,854 (7.1%) as infrequent, 27,933 (5.9%) as moderately frequent, and 150,326 (31.5%) as frequent OCS.

Because of the observed distribution of OCS use patterns, including the large proportion of mixed patterns, and for ease of interpretation, a simpler categorization was also used: **once-only** – only one prescription (*n* = 198,422); **less frequent** – sporadic, infrequent, moderately infrequent patterns or other mixed prescribing patterns with ≥90-day gap (*n* = 127,419); or **frequent** – patients with frequent or mixed prescribing patterns including frequent prescriptions (<90-day gap; *n* = 150,326). Patients with frequent OCS use were typically older, more likely to be female and on a longer period of baseline follow-up than those on less frequent and once-only OCS (mean age: 42.4 years, 36.9 years and 35.5 years, respectively; females: 60.2, 56.3, and 51.8%, respectively; median baseline follow-up [interquartile range]: 20.6 [9.1, 34.9] years, 16.2 [7.2, 30.1] years and 15.1 [6.9, 28.3] years, respectively). A detailed breakdown of the intermittent OCS use patterns is provided in Table 1 and the dosage per prescription is shown in Supplementary Fig. 2.

**Table 1:** Detailed analysis of the patterns of intermittent OCS use based on the spacing of OCS bursts and mean and median number of prescriptions used among UK patients with asthma.

OCS use pattern	*n* (%)	Mean number of prescriptions (SD)	Median number of prescriptions (IQR)
Once-only	198,422 (41.7)	1.0 (1.0)	1.0 (1.0, 1.0)
Sporadic	65,632 (13.8)	2.4 (0.9)	2.0 (2.0, 3.0)
Any Infrequent	33,854 (7.1)	3.1 (1.4)	3.0 (2.0, 4.0)
Infrequent only	1,445 (0.3)	2.2 (0.6)	2.0 (2.0, 2.0)
Sporadic and infrequent	32,409 (6.8)	3.1 (1.4)	3.0 (2.0, 4.0)
Any moderately frequent	27,933 (5.9)	3.7 (2.0)	3.0 (2.0, 4.0)
Moderately frequent only	155 (<0.1)	2.7 (0.9)	2.0 (2.0, 3.0)
Sporadic, infrequent and moderately frequent	8,836 (1.9)	5.3 (2.2)	5.0 (4.0, 6.0)
Sporadic and moderately frequent	17,371 (3.6)	3.0 (1.3)	3.0 (2.0, 4.0)
Infrequent and moderately frequent	1,571 (0.3)	2.6 (1.0)	2.0 (2.0, 3.0)
Any frequent	150,326 (31.5)	8.7 (13.3)	5.0 (3.0, 9.0)
Sporadic, infrequent, moderately frequent and frequent	39,842 (8.4)	16.2 (15.6)	12.0 (8.0, 19.0)
Sporadic, infrequent and frequent	29,432 (6.2)	6.8 (6.3)	6.0 (4.0, 8.0)
Sporadic, moderately frequent and frequent	17,455 (3.7)	8.4 (14.3)	6.0 (4.0, 8.0)
Sporadic and frequent	56,661 (11.9)	3.8 (5.3)	3.0 (2.0, 4.0)
Infrequent, moderately frequent and frequent	3,083 (0.6)	12.1 (20.0)	7.0 (4.0, 12.0)
Infrequent and frequent	2,136 (0.4)	4.9 (10.1)	3.0 (2.0, 5.0)
Moderately frequent and frequent	809 (0.2)	18.0 (26.6)	9.0 (6.0, 19.0)
Frequent only	908 (0.2)	39.4 (66.1)	20.0 (4.0, 46.0)
Total	476,167 (100.0)	3.9 (8.2)	2.0 (1.0, 4.0)

*IQR* interquartile range (25th–75th percentile), *OCS* oral corticosteroid, *SD* standard deviation.

### Intermittent OCS use by GINA treatment Step, SABA use and age

**Fig. 2:**
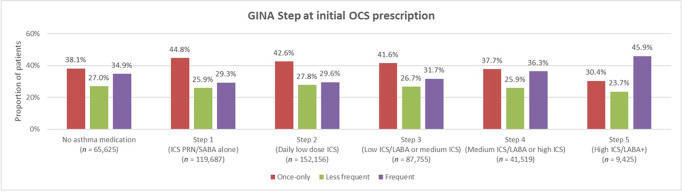
Intermittent OCS use by GINA Step at initial OCS prescription among UK patients with asthma. GINA Global Initiative for Asthma; ICS, inhaled corticosteroid; LABA, long-acting β_2_ agonist; OCS, oral corticosteroid; PRN, as-needed; SABA, short-acting β_2_ agonist. For GINA Step 5 treatment, ‘+’ refers to add-on therapy with e.g., tiotropium, anti-IgE, anti-IL5/5R or anti-IL4R therapies.

Of the 476,167 patients receiving intermittent OCS, 13.8% were on no asthma medication 12 months pre-index, 25.1% were on GINA Step 1 treatment, 32.0% on Step 2, 18.4% on Step 3, 8.7% on Step 4 and 2.0% on Step 5. Almost a third of patients with asthma receiving GINA Steps 1 and 2 treatments at the time of first OCS prescription had a frequent OCS use pattern at some point during follow-up. The increasing frequency pattern of intermittent OCS use was more likely to be observed in patients with greater asthma severity (GINA step; *p* < 0.05; Fig. 2) and those with ≥3 SABA fills (Table 2). The once-only OCS use pattern was more common in children and adolescents than in adults (46.0% and 53.3% vs 39.6%), with the frequent OCS use pattern more common in adults compared with children and adolescents (34.1% vs 24.7% and 20.4%) (Table 3).

**Table 2:** Intermittent OCS use by SABA use at initial OCS prescription among UK patients with asthma.

SABA prescriptions	Intermittent OCS use, *n* (%)			
	Any OCS (*n* = 476,167)	Once-only OCS (*n* = 198,422)	Less frequent OCS (*n* = 127,419)	Frequent OCS (*n* = 150,326)
0	113,262 (23.8)	44,766 (22.6)	30,987 (24.3)	37,507 (25.0)
1–2	213,594 (44.9)	96,751 (48.8)	55,660 (43.7)	61,183 (40.7)
≥3	149,311 (31.4)	56,903 (28.7)	40,772 (32.0)	51,636 (34.3)

*OCS* oral corticosteroid; *SABA* short-acting β_2_-agonist.

**Table 3:** Intermittent OCS use by age at initial OCS prescription among UK patients with asthma.

Age, years	Intermittent OCS use, *n* (%)			
	Any OCS (*n* = 476,167)	Once-only OCS (*n* = 198,422)	Less frequent OCS (*n* = 127,419)	Frequent OCS (*n* = 150,326)
≥4–<12	77,131 (16.2)	35,478 (46.0)	22,568 (29.3)	19,085 (24.7)
≥12–<18	36,262 (7.6)	19,336 (53.3)	9,518 (26.2)	7,408 (20.4)
≥18	362,774 (76.2)	143,608 (39.6)	95,333 (26.3)	123,833 (34.1)

*OCS* oral corticosteroid.

## Discussion

This historical cohort study in patients with asthma who only received intermittent OCS showed that although the largest proportion of studied patients had once-only OCS use (41.7%), almost a third of patients had a frequent pattern of use at some point during follow-up. Nearly a third of patients with mild asthma (GINA Steps 1–2) at first OCS prescription had a frequent pattern of intermittent OCS use, and the proportion of this frequent pattern of OCS use was higher in patients with greater asthma severity at baseline. Patients with more frequent pattern of OCS use were more likely to have a high number of SABA fills prior to their first OCS prescription.

Previous attempts to categorize OCS use were based on aggregate measures over specific but not universal time periods. Although cumulative OCS exposure provides insight into the use of OCS over time, it might not reflect variations in parameters such as disease duration and severity among patients^6^. We studied the prescribing patterns of intermittent (acute) OCS use post-index, as understanding how intermittent OCS is being used in asthma treatment is important for improving treatment and elucidating the relation between intermittent OCS use and risk of AEs. Compared with reporting OCS use by number of prescriptions alone, longitudinal patterns of OCS use could provide a more clinically relevant picture of the burden of intermittent treatment, taking into account changes in frequency over time. It should be noted, however, that the intermittent OCS patterns we have described were not intended to serve as a proxy for exacerbations. It is possible that some patients used the same prescription for multiple exacerbations, as suggested by Supplementary Fig. 1, which shows some prescriptions contained much higher doses than typical for a single exacerbation.

Analysis of OCS use by GINA treatment step in patients receiving intermittent OCS showed that a frequent pattern of intermittent OCS use was common even in mild and moderate asthma. This is supported by an analysis by Tran, et al.^13^ in which nearly 70% of US asthma patients with high OCS use during follow-up were classified with mild or moderate asthma at baseline. The observed link between higher asthma severity^14^ and worsening control^14–16^, and frequent pattern of OCS use suggest that this pattern of intermittent OCS use may reflect another aspect of OCS exposure (beside cumulative exposure) that might be important to understand. The finding that the frequent pattern of OCS use was more common in adults than adolescents and children is in line with previous studies in which OCS use (both maintenance and episodic) increased with age^5,17^. Further studies are also needed to explore the reported OCS prescribing patterns within this dataset in more granularity, as well as the impact of ICS-formoterol maintenance and reliever therapy on these patterns would also be interesting and important topics for future research.

Potential limitations of this study include the possible non-differential misclassification of some prescriptions, despite using a sophisticated algorithm. Moreover, by developing this algorithm, we focused on the specificity more than the sensitivity of exposure to OCS, which might have resulted in misidentifying some true intermittent OCS use. Finally, OCS prescriptions may not be directly linked to OCS use, as there is no guarantee that patients adhered to the prescriptions that they received, and it does not account for potential ‘stockpiling’ of OCS by patients.

In summary, our initial data showed that almost a third of patients with asthma who receive only intermittent OCS had a frequent pattern of use at some point, including those with mild asthma; patients with more severe asthma have a more frequent pattern of OCS use. This pattern-based approach may offer a clinically relevant representation of the burden of intermittent OCS therapy in asthma.

## Supplementary information

Supplementary Information

Reporting Summary

## Data Availability

The dataset supporting the conclusions of this article was derived from the Optimum Patient Care Research Database (www.opcrd.co.uk). The OPCRD has ethical approval from the National Health Service (NHS) Research Authority to hold and process anonymised research data (Research Ethics Committee reference: 15/EM/0150). This study was approved by the Anonymised Data Ethics Protocols and Transparency (ADEPT) committee – the independent scientific advisory committee for the OPCRD. The authors do not have permission to give public access to the study dataset; researchers may request access to OPCRD data for their own purposes. Access to OCPRD can be made via the OCPRD website (https://opcrd.co.uk/our-database/data-requests/) or via the enquiries email info@opcrd.co.uk. The dataset supporting the conclusions of this article was derived from the Optimum Patient Care Research Database. We do not have permission to give public access to this database, however, researchers may request access for their own purposes, in accordance with AstraZeneca’s data sharing policy described at https://astrazenecagrouptrials.pharmacm.com/ST/Submission/Disclosure.
