# Supplementary material for: Application of an algorithm to analyze patterns of intermittent oral corticosteroid use in asthma
Source: NPJ Prim Care Respir Med. 2023 Mar 4;33:9. doi: 10.1038/s41533-023-00331-0 (PMC9985594; doi:10.1038/s41533-023-00331-0)
Supplement: Supplementary file 1 — Supplementary Information [file 41533_2023_331_MOESM1_ESM.pdf]

**Supplementary Information**

**Supplementary Figure 1. Example patterns of frequent intermittent OCS use in two patients with asthma: (a) An adult and (b) a child aged 5 years at first OCS prescription.**

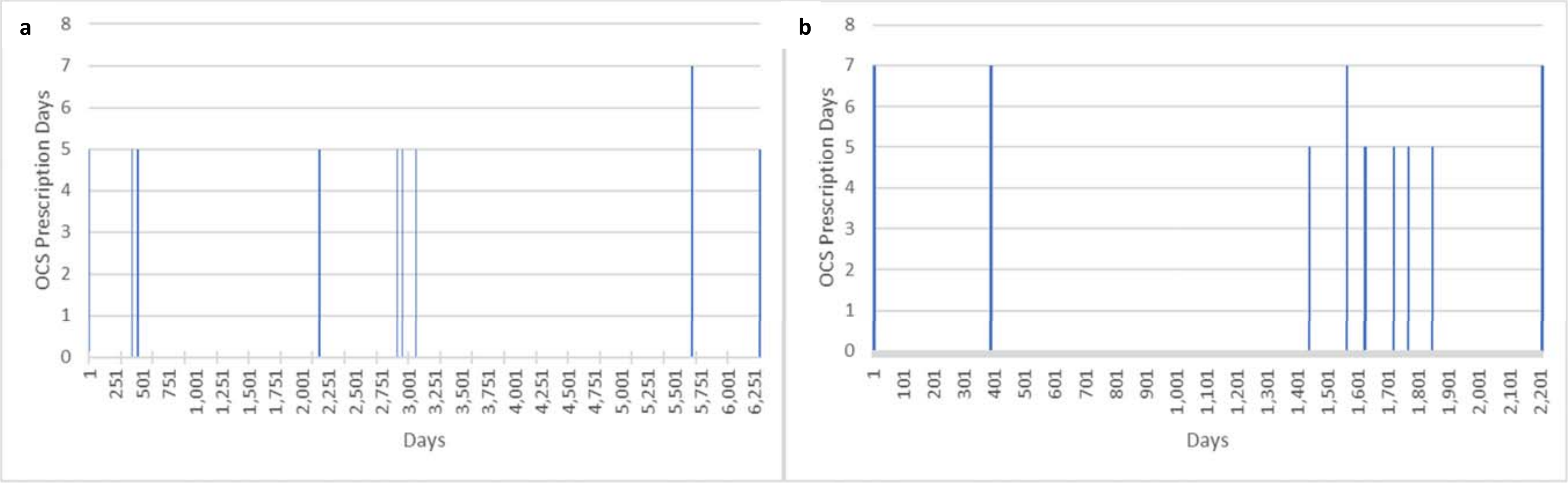


**OCS**, oral corticosteroid.

**Supplementary Figure 2. Intermittent OCS dosage per prescription among UK patients with asthma.**

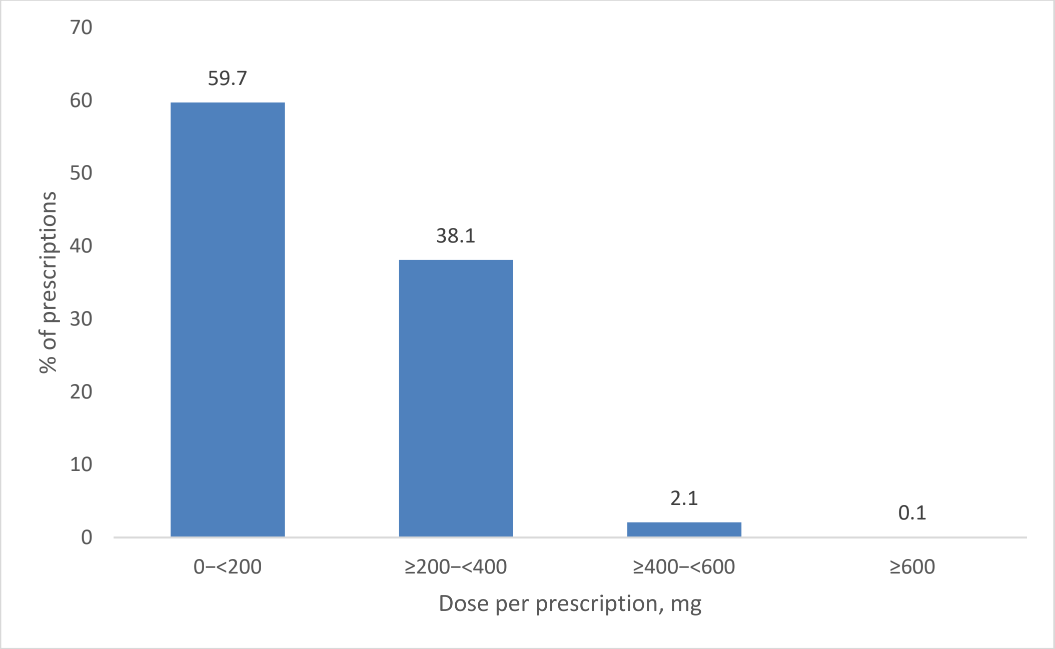


**OCS**, oral corticosteroid.
